# Emergence and transmission of New Delhi metallo‐beta‐lactamase‐5‐producing *Escherichia coli* Sequence Type 361 in a Tertiary Hospital in South Korea

**DOI:** 10.1002/jcla.23041

**Published:** 2019-09-20

**Authors:** Yumi Park, Qute Choi, Gye Cheol Kwon, Sun Hoe Koo

**Affiliations:** ^1^ Department of Laboratory Medicine Chungnam National University Hospital Daejeon Korea

**Keywords:** Carbapenemase‐producing *Enterobacteriaceae*, *Escherichia coli *ST 361, New Delhi metallo‐beta‐lactamase

## Abstract

**Background:**

The emergence of carbapenem‐resistant *Escherichia coli (E coli)* is a serious global health threat, but little is known about carbapenemase‐producing *E coli* in Daejeon, South Korea. The aim of this study was to investigate characteristics of thirteen carbapenem‐resistant *E coli* isolates in a tertiary hospital.

**Methods:**

Thirteen non‐duplicate carbapenem‐resistant *E coli* strains were collected from October 2017 to January 2018. Antimicrobial susceptibility was determined with the E test or disk diffusion method. The carbapenem minimum inhibitory concentrations (MICs) were determined by the agar dilution method. The colistin and tigecycline MICs were determined by broth microdilution. The resistance genes, including carbapenemase genes, were evaluated by polymerase chain reaction, and DNA sequencing was performed to characterize the genes. Pulsed‐field gel electrophoresis and multilocus sequence typing (MLST) were performed to evaluate the clonal relatedness of isolates. The clinical data of patients were retrospectively reviewed.

**Results:**

All the *E coli* isolates harbored *bla_NDM‐5_* gene and were resistant to most of the antimicrobial agents, such as carbapenem, cephalosporins, ciprofloxacin, and chloramphenicol, excluding amikacin and colistin. Other resistant genes, such as *bla_TEM‐1_*, *bla_CTX‐M‐15_*, *bla_CMY‐2_*, *aac(6')‐Ib‐cr*, and *qepA,* were detected. The *E coli* isolates harboring *bla*_NDM‐5_ belonged to ST361 (n = 11), ST12 (n = 1), ST410 (n = 1), and PFGE types A (n = 11), B (n = 1), and C (n = 1).

**Conclusions:**

This study reports on an outbreak of a predominant epidemic clone, the NDM‐5 producing, multidrug‐resistant *E coli* ST361 isolate. These findings suggest that we should pay attention to infection control measures to limit the spread of NDM‐5‐producing pathogens.

**Abbreviations**
AM: ampicillin
AMK: amikacin
ATM: aztreonam
CAZ: ceftazidime
CFZ: cefazolin
CHL: chloramphenicol
CIP: ciprofloxacin
CST: colistin
CTX: cefotaxime
ETP: ertapenem
FEP: cefepime
GEN: gentamicin
IPM: imipenem
ST: sequence typing
TGC: tigecycline
TMP/SMX: trimethoprim/sulfamethoxazole
TZP: piperacillin/tazobactam

## 1 INTRODUCTION

The *Enterobacteriaceae* family are leading causes of infections, such as urinary tract infections, hospital‐ and healthcare‐associated pneumonia, and bloodstream infections.^1^ Carbapenems that exhibit broad antibacterial activity among beta‐lactams are used as effective antibiotics against *Enterobacteriaceae‐*producing extended‐spectrum beta‐lactamases (ESBL) and plasmidic AmpC (pAmpC).^2^

However, as the emergence of carbapenemase‐producing *Enterobacteriaceae* (CPE) is increasingly reported worldwide, bacterial resistance to antibiotics has become a major source of concern for public health in recent years.^3^, ^4^, ^5^, ^6^ According to the report of the European Survey on carbapenemase‐producing *Enterobacteriaceae* (EuSCAPE), 19% (77/402) of *E coli* clinical isolates not susceptible to carbapenem, are carbapenemase producers^7^ (eg, KPC, NDM, VIM, or OXA‐48‐like). Carbapenemases mainly belong to Ambler classes A, B, and D of beta‐lactamase. Among them, New Delhi metallo‐beta‐lactamase (NDM) belongs to Ambler class B and can hydrolyze almost all beta‐lactam antibiotics, including penicillins, cephalosporins, and carbapenems, except monobactams, such as aztreonam.^8^, ^9^

Since NDM‐1 producing *Klebsiella pneumonia* isolates were initially described in 2008,^9^ to date, 21 NDM subtype variants have been reported worldwide.^10^ The NDM subtypes contain one to five amino‐acid substitutions that confer different levels of hydrolyzing activity against carbapenems and other β‐lactam substrates.^3^

Among NDM variants, the first NDM‐5 was identified in 2011 from a multidrug‐resistant *E coli* ST648 isolate in the United Kingdom, from a patient previously hospitalized in India, and differed from NDM‐1 with two amino acid substitutions at positions 88 (Val → Leu) and 54 (Met → Leu).^11^

In South Korea, the *bla_NDM‐5_* gene was first detected in 2015, in *Klebsiella pneumoniae* clinical isolates co‐producing oxacillinase 181,^12^ and has been reported intermittently since then. However, an outbreak of NDM‐5‐producing isolates, especially *E coli* ST 361, has not yet been described.

In this study, we report an outbreak of NDM‐5‐producing *E coli* in a tertiary hospital in South Korea and characterize the molecular epidemiology and antibiotic resistance profiles of the isolates.

## 2 MATERIALS AND METHODS

### 2.1 Bacterial strains

Chungnam National University Hospital is a 1300‐bed tertiary care hospital located in the Daejeon of South Korea, a city of about 1 490 000 residents. Prior to this outbreak, carbapenem‐resistant *E coli* isolates were rarely detected and no case involving carbapenemase‐producing *E coli* had been detected in this hospital.

CR‐ECO1, which is resistant to carbapenem, including ertapenem and imipenem, was isolated from a urine specimen, obtained from a patient in the neurosurgery ward on October 25, 2017. On October 31, 2017, another carbapenem‐resistant CR‐ECO2 was isolated from a urine sample of a patient hospitalized in the same ward, within the same period. Until January 23, 2018, a total of thirteen non‐duplicate carbapenem‐resistant *E coli* isolates were collected in the hospital. The identification of isolates was performed using the VITEK 2 ID‐GNB cards (bioMérieux SA) according to the manufacturer instructions. The modified Hodge test, using ertapenem disks and Carba NP (bioMérieux SA), was conducted to confirm the phenotypic identification of carbapenemase production.^13^ Both *E coli* ATCC 25922 and *Pseudomonas aeruginosa* ATCC 27853 were used as quality control strains for antimicrobial susceptibility testing. *Salmonella enterica serovar Braenderup* strain H9812 (ATCC BAA 664) was used as a reference marker for pulsed‐field gel electrophoresis (PFGE). The clinical data for each patient were retrospectively reviewed.

### 2.2 Antimicrobial susceptibility testing

The minimum inhibitory concentrations (MICs) of the carbapenems, such as ertapenem and imipenem, were determined by the agar dilution method, according to the Clinical and Laboratory Standards Institute (CLSI) guideline.^14^ Antimicrobial susceptibility to nine drugs (piperacillin/tazobactam, cefepime, cefotaxime, ceftazidime, gentamicin, amikacin, ciprofloxacin, trimethoprim/sulfamethoxazole, and chloramphenicol) was evaluated using the E test (bioMérieux) on Mueller‐Hinton (MH) agar (Difco Laboratories) in accordance with CLSI guidelines.^15^ Antimicrobial susceptibility to three drugs (ampicillin, cefazolin, and aztreonam) was evaluated using the disk diffusion method on MH agar (Difco Laboratories) in accordance with CLSI guidelines.^15^ The MICs for colistin and tigecycline were assessed by the broth microdilution method with MH broth (Difco Laboratories) in accordance with the recommendations of the joint CLSI–EUCAST (2016)^16^ and European Committee on Antimicrobial Susceptibility Testing (EUCAST) criteria.^17^

### 2.3 Resistance gene detection

Bacterial DNA was extracted using an ExiPrep Dx Bacteria Genomic DNA Kit (BIONEER) according to the manufacturer instructions. The carbapenemase genes (*bla_NDM_*, *bla_IMP_*, *bla_VIM_*, *bla_KPC_*, *bla_OXA‐48‐like_*_,_ *bla_GES_*, and *bla_OXA‐181_*) were detected by PCR and direct sequencing was carried out for subtyping.^18^ The presence of other resistance genes: (a) extended spectrum‐β‐lactamases (ESBLs) encoding genes (*bla_CTX−M−1−like_*, *bla_CTX−M−9−like_*, *bla_TEM_*, and *bla_SHV_*); (a) AmpC genes (*bla_CIT_*, *bla_MOX_*, *bla_DHA_*, *bla_ACC_*, *bla_EBC_*, and *bla_FOX_*); (c) plasmid‐mediated quinolone resistance genes, DNA gyrase protection from the action of the quinolones (*qnrA*, *qnrB*, and *qnrS*), antibiotics acetylation (*aac(6')‐Ib‐cr*), and efflux pump production (*qepA*); and (d) 16S ribosomal methyltransferases (*armA*, *rmtA*, *rmtB*, and *rmtC*)^19^, ^20^ was detected by PCR with gene‐specific primers. The amplicons were determined by DNA sequencing. The primers for the PCR are shown in Table 1.

**Table 1:** The primers used to detect the resistance genes

	Gene	Primer	Sequence (5′—3′)	Size of amplicon/bp	Reference
Carbapenem	*bla_NDM_*	F R	GGTTTGGCGATCTGGTTTTC CGGAATGGCTCATCACGATC	621	18
	*bla_IMP_*	F R	GGAATAGAGTGGCTTAAYTC GGTTTAAYAAAACAACCACC	232	
	*bla_VIM_*	F R	GATGGTGTTTGGTCGCATA CGAATGCGCAGCACCAG	390	
	*bla_KPC_*	F R	CGTCTAGTTCTGCTGTCTTG CTTGTCATCCTTGTTAGGCG	798	
	*bla_OXA‐48_*	F R	GCGTGGTTAAGGATGAACAC CATCAAGTTCAACCCAACCG	438	
	*bla_GES_*	F R	GCTTCATTCACGCACTATT CGATGCTAGAAACCGCTC	323	28
	*bla_OXA‐181_*	F R	ATGCGTGTATTAGCCTTATCG AACTACAAGCGCATCGAGCA	888	29
β‐lactamase	*bla_CTX‐M‐1_*	F R	AGTTCACGCTGATGGCGACG AACCCAGGAAGCAGGCAGTCC	676	30
	*bla_CTX‐M‐9_*	F R	GATTGACCGTATTGGGAGTTT CGGCTGGGTAAAATAGGTCA	947	31
	*bla_TEM_*	F R	ATAAAATTCTTGAAGACGAA GACAGTTACCAATGCTTAAT	1080	32
	*bla_shv_*	F R	GGGTTATTCTTATTTGTCGC TTAGCGTTGCCAGTGCTC	928	
AmpC	*bla_cit_*	F R	TGGCCAGAACTGACAGGCAAA TTTCTCCTGAACGTG GCTGGC	462	19
	*bla_mox_*	F R	GCTGCTCAAGGAGCACAGGAT CACATTGACATAGGTGTGGTGC	520	
	*bla_dha_*	F R	AACTTTCACAGGTGTGCTGGGT CCGTACGCATACTGGCTTTGC	405	
	*bla_acc_*	F R	AACAGCCTCAGCAGCCGGTTA TTCGCCGCAATCATCCCTAGC	346	
	*bla_ebc_*	F R	TCGGTAAAGCCGATGTTGCGG CTTCCACTGCGGCTGCCAGTT	302	
	*bla_FOX_*	F R	AACATGGGGTATCAGGGAGATG CAAAGCGCGTAACCGGATTGG	190	
Quinolone	*qnrA*	F R	AGAGGATTTCTCACGCCAGG TGCCAGGCACAGATCTTGAC	580	33
	*qnrB*	F R	GGMATHGAAATTCGCCACTG^a^ TTTGCYGYYCGCCAGTCGAA^a^	264	
	*qnrS*	F R	GCAAGTTCATTGAACAGGGT TCTAAACCGTCGAGTTCGGCG	428	
	*aac(6')‐Ib‐cr*	F R	TGACCAACAGCAACGATTCC TTAGGCATCACTGCGTGTTC	554	34
	*qepA*	F R	GGACATCTACGGCTTCTTCG AGCTGCAGGTACTGCGTCAT	720	35
16S rRNA methylase	*armA*	F R	AGGTTGTTTCCATTTCTGAG TCTCTTCCATTCCCTTCTCC	591	36
	*rmtA*	F R	CTAGCGTCCATCCTTTCCTC TTTGCTTCCATGCCCTTGCC	635	
	*rmtB*	F R	CCCAAACAGACCGTAGAGGC CTCAAACTCGGCGGGCAAGC	585	
	*rmtC*	F R	CGAAGAAGTAACAGCCAAAG ATCCCAACATCTCTCCCACT	711	

Abbreviations: F, forward; R, reverse.

a M = A or C; H = A or C or T; Y = C or T.

### 2.4 Pulsed‐field gel electrophoresis (PFGE) and multilocus sequence typing (MLST)

PFGE and MLST were used to investigate the homology levels among the *bla_NDM‐5_*‐positive *E coli* isolates. Bacterial DNA was prepared and cleaved with XbaI endonuclease (Roche) as described previously.^21^ The XbaI‐digested genomic DNA was subjected to PFGE using a CHEF‐DR^®^ III Variable Angle System (Bio‐Rad), and then the PFGE patterns were compared using BioNumerics software (Applied Maths). Clusters were defined as DNA patterns sharing >85% similarity. PCR and sequencing for MLST were carried out for seven housekeeping genes per species: *adk*, *fumC*, *gyrB*, *icd*, *mdh*, *purA*, and *recA* for *E coli* 22 and the sequences were compared in the MLST database, so that allelic numbers and sequence types (STs) could be determined.^3^ The allelic profiles and STs were assigned using an online database (http://mlst.warwick.ac.uk/mlst/dbs/Ecoli).

## 3 RESULTS

### 3.1 Clinical characteristics of the *Escherichia coli* isolates

**Figure 1:**
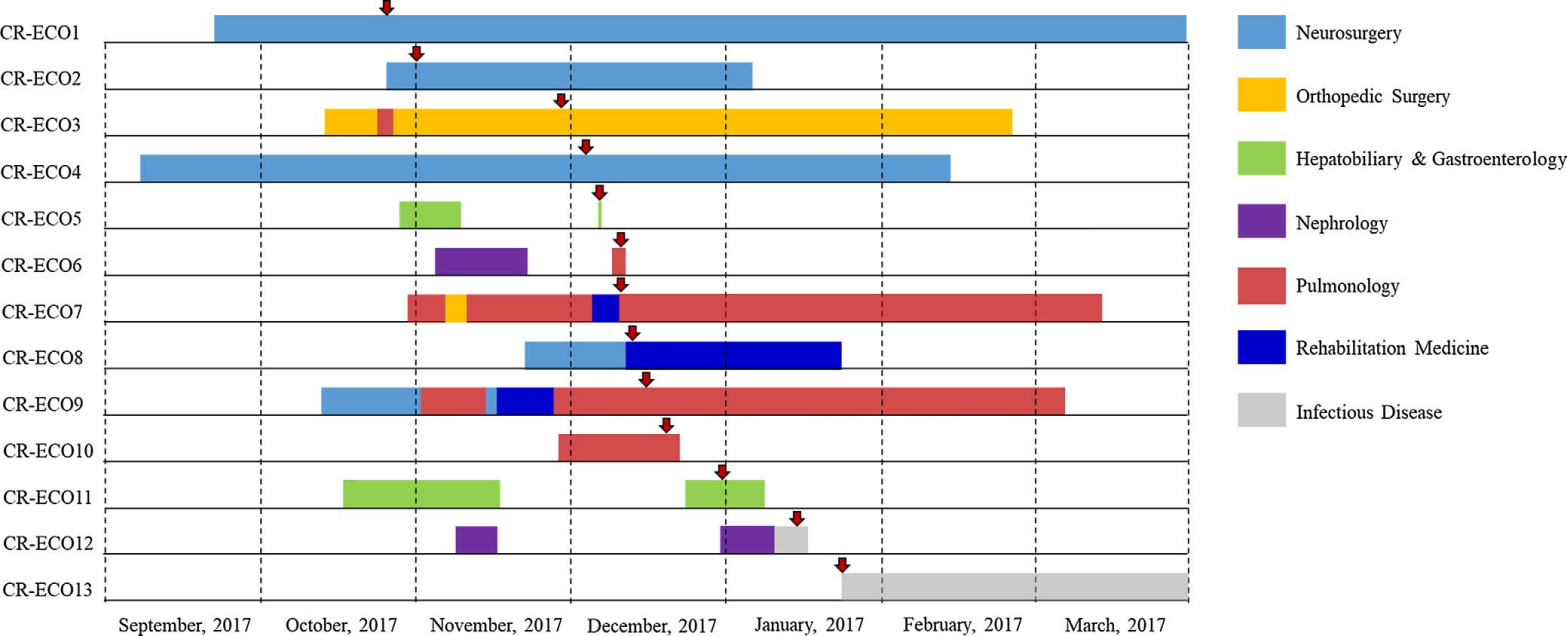
Outbreak timeline of thirteen patients with bla_NDM‐5_‐producing *Escherichia coli* isolates. Shadows on the timeline represent the period of hospitalization and the wards. Shadows in different colors indicate different wards. The red arrow indicates the isolation date of the strains

All thirteen carbapenem‐resistant *E coli* isolates showed positive phenotypic screening results for both the Modified Hodge test and the Carba‐NP test. These isolates were obtained from stool (n = 6), urine (n = 5), bile fluid (n = 1), and pus (n = 1) from hospitalized patients aged from 46 to 83, with mean age of 69 years (Table 2). The stool samples were obtained using rectal swabs from patients admitted to an intensive care unit for CPE screening test. The outbreak timeline on the admission date and date of isolation of each patient is shown in Figure 1.

**Table 2:** Characteristics of *bla_NDM‐5_*‐positive *Escherichia coli* isolates

Isolate No.	Age (y)/sex	Date of isolation	Specimen	Diagnosis
CR‐ECO 1	56/M	10/25/2017	Urine	Traumatic subdural hemorrhage
CR‐ECO 2	83/F	10/31/2017	Urine	Spinal stenosis
CR‐ECO 3	73/F	11/29/2017	Urine	Fracture of shaft of femur
CR‐ECO 4	61/M	12/03/2017	Stool	Hydrocephalus
CR‐ECO 5	75/M	12/05/2017	Bile fluid	Malignant neoplasm of gallbladder
CR‐ECO 6	76/F	12/10/2017	Stool	Pneumonia
CR‐ECO 7	82/F	12/10/2017	Urine	Pneumonia
CR‐ECO 8	62/F	12/12/2017	Stool	Subarachnoid hemorrhage
CR‐ECO 9	78/F	12/15/2017	Urine	Cervical myelopathy & pneumonia
CR‐ECO 10	78/F	12/19/2017	Stool	Pneumonia
CR‐ECO 11	53/F	12/30/2017	Pus	Ulcerative colitis
CR‐ECO 12	75/M	01/14/2018	Stool	Chronic kidney disease
CR‐ECO 13	46/M	01/23/2018	Stool	Meningitis

### 3.2 Antibiotic resistance profile and distribution of resistant genes

**Figure 2:**
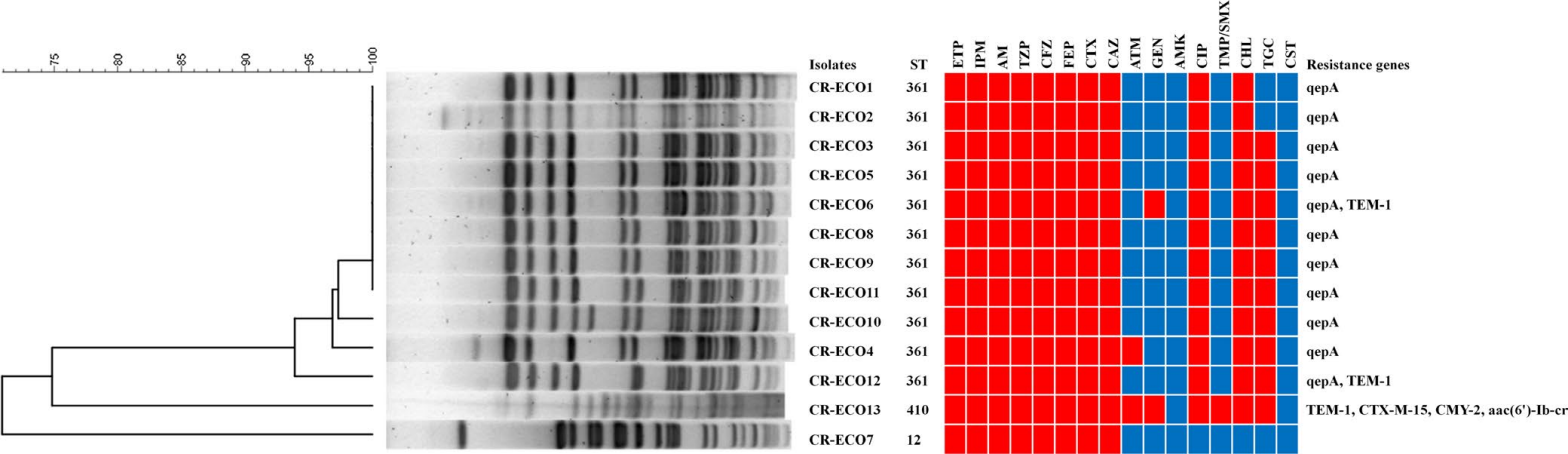
Dendrogram based on pulsed‐field gel electrophoresis patterns, multilocus sequence typing, antibiogram, and distribution of resistance genes of thirteen NDM‐5‐producing *Escherichia coli* isolates. The red and blue squares indicate resistance and susceptibility to each antibiotic, respectively

All thirteen *E coli* isolates harbored *bla_NDM‐5_* gene and were resistant to carbapenem (ertapenem and imipenem), ampicillin, piperacillin/tazobactam, and cephalosporins (cefazolin, cefepime, cefotaxime, and ceftazidime; Table 3). These isolates were also resistant to ciprofloxacin (92.3%, 12/13), chloramphenicol (92.3%, 12/13), tigecycline (76.9%, 10/13), gentamicin (15.4%, 2/13), and trimethoprim/sulfamethoxazole (7.7%, 1/13). Two isolates out of thirteen were resistant to aztreonam (15.4%, 2/13). All strains remained susceptible to colistin and amikacin. In addition to *bla_NDM‐5_*, 11/13 strains harbored the *qepA* gene (Figure 2). The *bla_TEM‐1_* was also detected in 23.1% (3/13). In particular, CR‐ECO13 isolates also co‐harbored *bla_CMY‐2_*, *bla_CTX‐M‐15_*, and *aac(6)–Ib‐cr* genes, as well as *bla_TEM‐1_*. Other resistant genes that were evaluated, such as *qnrA*, *qnrB*, *qnrS*, *armA*, *rmtA*, *rmtB*, and *rmtC*, were not detected.

**Table 3:** Antibiotic susceptibilities of 13 bla_NDM‐5_‐positive *Escherichia coli* isolates

Isolate No.	MICs (µg/mL)												
	ETP	IPM	TZP	FEP	CTX	CAZ	GEN	AMK	CIP	TMP/SMX	CHL	TGC	CST
CR‐ECO1	32	64	>128	>256	>256	>256	1	2	>32	0.125	>256	0.5	2
CR‐ECO2	32	64	>128	>256	>256	>256	1	2	>32	0.125	>256	0.5	2
CR‐ECO3	16	8	>128	>256	>256	>256	1	2	>32	0.19	>256	1	0.5
CR‐ECO4	32	128	>128	>256	>256	>256	0.125	0.125	>32	0.25	>256	1	0.5
CR‐ECO5	32	128	>128	>256	>256	>256	1	2	>32	0.25	>256	1	0.5
CR‐ECO6	32	64	>128	>256	>256	>256	32	1	>32	0.5	>256	1	2
CR‐ECO7	32	64	>128	64	>256	>256	1	2	0.016	0.064	2	0.5	1
CR‐ECO8	32	256	>128	>256	>256	>256	1	1.5	>32	0.125	>256	1	2
CR‐ECO9	32	64	>128	>256	>256	>256	1	2	>32	0.38	>256	4	2
CR‐ECO10	32	256	>128	>256	>256	>256	1	3	>32	0.125	>256	1	2
CR‐ECO11	32	64	>128	>256	>256	>256	1	1	>32	0.19	>256	1	2
CR‐ECO12	32	128	>128	>256	>256	>256	1	1	>32	0.19	>256	1	2
CR‐ECO13	32	128	>128	>256	>256	>256	64	8	>32	>32	>256	1	0.5

Abbreviations: AMK, amikacin (0.016‐256 µg/mL); CAZ, ceftazidime (0.016‐256 µg/mL); CHL, chloramphenicol (0.016‐256 µg/mL); CIP, ciprofloxacin (0.002‐32 µg/mL); CST, colistin; TMP/SMX, trimethoprim/sulfamethoxazole (0.002‐32 µg/mL); CTX, cefotaxime (0.016‐256 µg/mL); ETP, ertapenem; FEP, cefepime (0.016‐256 µg/mL); GEN, gentamicin (0.064‐1024 µg/mL); IPM, imipenem; MICs, minimum inhibitory concentrations; TGC, tigecycline; TZP, piperacillin/tazobactam (0.016‐256 µg/mL).

### 3.3 Molecular epidemiology by MLST & PFGE

Three distinct MLST STs were observed among the thirteen isolates: ST361 with allelic profile 10‐99‐5‐91‐8‐7‐2 (11/13, 84.6%), ST12 (1/13, 7.7%), and ST410 (1/13, 7.7%). Moreover, three distinct PFGE patterns (PFGE types A‐C) were observed among these thirteen isolates: type A (n = 11), type B (n = 1), and type C (n = 1; Figure 1). Comparison of these results showed that all PFGE type A isolates corresponded to ST361, and the type B and C isolates corresponded to ST12 and ST410, respectively.

## 4 DISCUSSION

In this study, we reported and characterized an outbreak caused by thirteen *bla_NDM‐5_* carrying *E coli* isolates from hospitalized patients from October 2017 to January 2018. The *bla_NDM‐5_* gene played an important role in conferring resistance to carbapenem. PFGE analysis showed that eleven out of thirteen isolates exhibited ≥90% similarities and belonged to the ST361 epidemic clone. Among them, eight isolates had the same pulsotype in PFGE, indicating they were clonally similar and showed a similar resistance phenotype in the antibiotic susceptibility testing. This finding suggests the possibility of nosocomial cross‐transmission.

The predominant strain of this outbreak, *E coli *ST361, is not internationally well‐known as an NDM producer. According to Yoon et al,^3^ the distribution of NDM‐5 producers in South Korea from 2010 to 2015 shows that *E coli* is the largest (14/18, 77.8%) and among them, ST 101 accounted for 77.8% (9/14), followed by ST362, ST361, ST162, ST90, and ST88 each accounted for 7.1% (1/14). The *E coli* ST361 harboring *bla_NDM‐5_* only accounted for 7.1% (1/14) and did not belong to the most common clones, such as ST101, in South Korea. Therefore, this is the first outbreak report involving NDM‐5‐producing *E coli* ST361.

Here, the NDM‐5‐producing *E coli* ST361 strains showed high levels of multidrug resistance to carbapenem (ertapenem and imipenem), ampicillin, piperacillin/tazobactam, cephalosporins (cefazolin, cefepime, cefotaxime, and ceftazidime), ciprofloxacin, and chloramphenicol. Among the plasmid‐mediated quinolone resistance genes, the co‐harbored *qepA* genes encoding fluoroquinolone efflux pumps could partially contribute to the high rate of ciprofloxacin‐resistance.^23^ All ST361 strains were susceptible to amikacin, colistin, and trimethoprim/sulfamethoxazole, which mean they are treatment options with potent activity against these pathogens*.* A few cases of NDM‐5‐producing *E *coli ST361 have also been reported in China^24^, ^25^ and Nepal.^26^ The *bla_NDM‐5_*_‐_carrying *E coli* ST361 that was isolated in Henan, China, showed resistance to sulfamethoxazole and co‐harbored various resistance genes such as *bla_CTX‐M‐15_*, *bla_TEM‐1_*, *aac(6)–Ib–cr*, and *qnrs*.^25^ Additionally, the *bla_NDM‐5_*‐carrying *E coli* ST361 that was isolated in Zhejiang province, China carried *bla_CMY‐42_* and *bla_TEM‐1B_* via an IncX3 type plasmid.^24^ However, resistance genes such as ESBLs, ampC genes, 16s ribosomal methyltransferases, and quinolone resistance genes, except for *qepA*, were not detected in NDM‐5‐producing *E coli* ST361 in this study.

The CR‐ECO 13 isolate, which is *E coli* ST410, is an extensively drug‐resistant strain, resistant to almost all antibiotics except for amikacin and colistin. This strain co‐harbored *bla_CTX‐M‐15_*, *bla_CMY‐2_*, and *bla_TEM‐1_*, which encodes an ESBL, conferring resistance to aztreonam and *aac(6')‐Ib‐cr*, which mediates high‐level resistance to aminoglycosides and fluoroquinolones. *Escherichia coli* ST410 has been reported worldwide as a potential high‐risk pathogen associated with resistance to fluoroquinolones, third generation cephalosporins, and carbapenems.^27^ Although ST410 is not a main strain of this outbreak, it poses a significant public health risk. These findings suggest that strict infection control is essential to prevent a dissemination of these high‐risk clones. The *E coli* ST12 (CR‐ECO 7) was susceptible to various antimicrobial agents (eg, aztreonam, gentamicin, amikacin, ciprofloxacin, trimethoprim/sulfamethoxazole, chloramphenicol, and colistin) and negative for other resistant genes tested, except for *bla_NDM‐5_*.

We carried out retrospective investigation with limited patient information. So, the entry and transmission route of *bla_NDM‐5_*‐producing *E coli* in this hospital is unclear. Environmental culture tests were carried out to investigate the possibility of dissemination through medical equipment and the surrounding environment, but carbapenemase‐producing *E coli* were not detected. Other patients in contact with known NDM‐5 carriers were also screened, but no NDM‐5 producers were detected. However, PFGE patterns showed that ST361 isolates were closely related. Also, some patients admitted to the same ward during the same period, overlapped with each other. Therefore, we suspect that NDM‐5‐producing *E coli* was transmitted by patients or medical staff. The relatively long hospitalization period of patients may also increase the possibility of dissemination of the NDM‐5‐producing *E coli* isolates. To prevent further spread, enhanced infection control measures, such as strengthening of hand hygiene, contact precaution, environmental cleaning, and preemptive patient isolation, were implemented. The outbreak was interrupted in February 2018, 3 months after isolation of the first NDM‐5‐producing *E coli*.

Considering that *E coli* is one of the major causes of community‐acquired infections, and the spread of these strains is possible not only in the hospital, but also in the surrounding environment, the severity of dissemination of *E coli* carrying the *bla_NDM‐5_* gene will be even greater.^2^

In conclusion, the rapid dissemination of NDM‐5‐producing *E coli* emphasizes that stringent infection control measures and active surveillance play important roles to prevent the spread of these pathogens.

## CONFLICTS OF INTEREST

The authors declare that there is no conflict of interest regarding the publication of this article.

## References

[1] Paterson DL . Resistance in gram‐negative bacteria: Enterobacteriaceae. Am J Infect Control. 2006;34(Suppl 5):20‐28. 10.1016/j.ajic.2006.05.238 16813978

[2] Meletis G . Carbapenem resistance: overview of the problem and future perspectives. Ther Adv Infect Dis. 2016;3:15‐21. 26862399 10.1177/2049936115621709 PMC4735501

[3] Yoon E‐J , Kang DY , Yang JW , et al. New Delhi Metallo‐Beta‐Lactamase‐Producing Enterobacteriaceae in South Korea Between 2010 and 2015. Front Microbiol. 2018;9:571. 29651277 10.3389/fmicb.2018.00571 PMC5884925

[4] Struelens MJ , Monnet DL , Magiorakos AP , Santos O'Connor F , Giesecke J ; the European NDM‐1 Survey Participants . New Delhi metallo‐beta‐lactamase 1–producing Enterobacteriaceae: emergence and response in Europe. Eurosurveillance. 2010;15(46):19716. 21144431 10.2807/ese.15.46.19716-en

[5] Nordmann P . Carbapenemase‐producing Enterobacteriaceae: overview of a major public health challenge. Med Mal Infect. 2013;44:51‐66. 24360201 10.1016/j.medmal.2013.11.007

[6] Albiger B , Glasner C , Struelens MJ , Grundmann H , Monnet DL . Carbapenemase‐producing Enterobacteriaceae in Europe: assessment by national experts from 38 countries. Eurosurveillance. 2015;20: 30062. 10.2807/1560-7917.ES.2015.20.45.30062 26675038

[7] Grundmann H , Glasner C , Albiger B , et al. Occurrence of carbapenemase‐producing Klebsiella pneumoniae and Escherichia coli in the European survey of carbapenemase‐producing Enterobacteriaceae (EuSCAPE): a prospective, multinational study. Lancet Infect Dis. 2017;17:153‐163. 27866944 10.1016/S1473-3099(16)30257-2

[8] Khan AU , Maryam L , Zarrilli R . Structure, genetics and worldwide spread of New Delhi Metallo‐β‐lactamase (NDM): a threat to public health. BMC Microbiol. 2017;17:101. 28449650 10.1186/s12866-017-1012-8 PMC5408368

[9] Yong D , Toleman MA , Giske CG , et al. Characterization of a New Metallo‐β‐Lactamase Gene, bla(NDM‐1), and a novel erythromycin esterase gene carried on a unique genetic structure in klebsiella pneumoniae sequence type 14 from India. Antimicrob Agents Chemother. 2009;53:5046‐5054. 19770275 10.1128/AAC.00774-09 PMC2786356

[10] Liu L , Feng Y , McNally A , Zong Z . blaNDM‐21, a new variant of blaNDM in an Escherichia coli clinical isolate carrying blaCTX‐M‐55 and rmtB. J Antimicrob Chemother. 2018;73:2336‐2339. 29912337 10.1093/jac/dky226

[11] Hornsey M , Phee L , Wareham DW . A Novel Variant, NDM‐5, of the New Delhi Metallo‐β‐Lactamase in a Multidrug‐Resistant Escherichia coli ST648 isolate recovered from a patient in the United Kingdom. Antimicrob Agents Chemother. 2011;55:5952‐5954. 21930874 10.1128/AAC.05108-11 PMC3232805

[12] Cho SY , Huh HJ , Baek JY , et al. Klebsiella pneumoniae Co‐Producing NDM‐5 and OXA‐181 Carbapenemases, South Korea. Emerg Infect Dis. 2015;21:1088‐1089. 25988911 10.3201/eid2106.150048 PMC4451906

[13] Clinical and Laboratory Standards Institute . CLSI M100. Performance standards for antimicrobial susceptibility testing. Available from. 2017; https://clsi.org/standards/products/microbiology/documents/m100/

[14] Clinical and Laboratory Standards Institute . Methods for dilution antimicrobial susceptibility tests for bacteria that grow aerobically. Wayne, PA: Clinical and Laboratory Standards Institute; 2015.

[15] Clinical and Laboratory Standards Institute . Performance standards for antimicrobial susceptibility testing; twenty‐fifth informational supplement. Wayne, PA: Clinical and Laboratory Standards Institute; 2015.

[16] The joint CLSI–EUCAST, and Polymyxin Breakpoints Working Group . Recommendations for MIC Determination of Colistin (polymyxin E) as Recommended by the Joint CLSI–EUCAST Polymyxin Breakpoints Working Group. 2016 Available from: http://www.eucast.org/fileadmin/src/media/PDFs/EUCAST_files/General_documents/Recommendations_for_MIC_determination_of_colistin_March_2016.pdf

[17] European Committee on Antimicrobial Susceptibility Testing (EUCAST) . Clinical breakpoints for bacteria. Available from: http://www.eucast.org/fileadmin/src/media/PDFs/EUCAST_files/Breakpoint_tables/v_9.0_Breakpoint_Tables.pdf

[18] Poirel L , Walsh T , Cuvillier V , Nordmann P . Multiplex PCR for detection of acquired carbapenemase genes. Diagn Microbiol Infect Dis. 2011;70(1):119‐123. 21398074 10.1016/j.diagmicrobio.2010.12.002

[19] Pérez‐Pérez FJ , Hanson ND . Detection of plasmid‐mediated AmpC β‐lactamase genes in clinical isolates by using multiplex PCR. J Clin Microbiol. 2002;40:2153‐2162. 12037080 10.1128/JCM.40.6.2153-2162.2002 PMC130804

[20] Ryoo NH , Kim E‐C , Hong SG , et al. Dissemination of SHV‐12 and CTX‐M‐type extended‐spectrum β‐lactamases among clinical isolates of Escherichia coli and Klebsiella pneumoniae and emergence of GES‐3 in Korea. J Antimicrob Chemother. 2005;56:698‐702. 16141280 10.1093/jac/dki324

[21] Qin S , Fu Y , Zhang Q , et al. High incidence and endemic spread of NDM‐1‐positive enterobacteriaceae in Henan province, China. Antimicrob Agents Chemother. 2014;58:4275‐4282. 24777095 10.1128/AAC.02813-13 PMC4136005

[22] Wirth T , Falush D , Lan R , et al. Sex and virulence in Escherichia coli: an evolutionary perspective. Mol Microbiol. 2006;60:1136‐1151. 16689791 10.1111/j.1365-2958.2006.05172.x PMC1557465

[23] Yamane K , Wachino J‐I , Suzuki S , et al. New plasmid‐mediated fluoroquinolone efflux pump, QepA, found in an Escherichia coli clinical isolate. Antimicrob Agents Chemother. 2007;51:3354‐3360. 17548499 10.1128/AAC.00339-07 PMC2043241

[24] Li XI , Fu Y , Shen M , et al. Dissemination of blaNDM‐5 gene via an IncX3‐type plasmid among non‐clonal Escherichia coli in China. Antimicrob Resist Infect Control. 2018;7:59. 29713466 10.1186/s13756-018-0349-6 PMC5918551

[25] Liang W‐J , Liu H‐Y , Duan G‐C , Zhao Y‐X , Chen S‐Y , Yang H‐Y . Emergence and mechanism of carbapenem‐resistant Escherichia coli in Henan, China, 2014. J Infect Public Health. 2018;11(3):347‐351. 29107607 10.1016/j.jiph.2017.09.020

[26] Shrestha B , Tada T , Shimada K , et al. Emergence of various NDM‐Type‐Metallo‐β‐lactamase‐producing escherichia coli clinical isolates in Nepal. Antimicrob Agents Chemother. 2017;61:e01425‐e11417. 28993336 10.1128/AAC.01425-17 PMC5700343

[27] Roer L , Overballe‐Petersen S , Hansen F , et al. Escherichia coli sequence type 410 is causing New International High‐Risk Clones. mSphere. 2018;3:e00337‐e1318. 30021879 10.1128/mSphere.00337-18 PMC6052333

[28] Hong SS , Kim K , Huh JY , Jung B , Kang MS , Hong SG . Multiplex PCR for rapid detection of genes encoding class A carbapenemases. Ann Lab Med. 2012;32:359‐361. 22950072 10.3343/alm.2012.32.5.359 PMC3427824

[29] Shanthi M , Sekar U , Arunagiri K , Bramhne HG OXA‐181 Beta Lactamase is not a major mediator of carbapenem resistance in Enterobacteriaceae. J Clin Diagn Res. 2013;7:1986‐1988. 24179916 10.7860/JCDR/2013/5884.3379 PMC3809655

[30] Bogaerts P , Galimand M , Bauraing C , et al. Emergence of ArmA and RmtB aminoglycoside resistance 16S rRNA methylases in Belgium. J Antimicrob Chemother. 2007;59:459‐464. 17224412 10.1093/jac/dkl527

[31] Kim MH , Lee HJ , Park KS , Suh JT . Molecular characteristics of extended spectrum beta‐lactamases in Escherichia coli and Klebsiella pneumoniae and the prevalence of qnr in Extended spectrum beta‐lactamase isolates in a tertiary care hospital in Korea. Yonsei Med J. 2010;51:768‐774. 20635454 10.3349/ymj.2010.51.5.768 PMC2908884

[32] Yao F , Qian Y , Chen S , Wang P , Huang Y . Incidence of extended‐spectrum β‐Lactamases and characterization of integrons in extended‐spectrum β‐Lactamase‐producing Klebsiella pneumoniae isolated in Shantou, China. Acta Biochim Biophys Sin. 2007;39:527‐532. 17622472 10.1111/j.1745-7270.2007.00304.x

[33] Cattoir V , Poirel L , Rotimi V , Soussy C‐J , Nordmann P . Multiplex PCR for detection of plasmid‐mediated quinolone resistance qnr genes in ESBL‐producing enterobacterial isolates. J Antimicrob Chemother. 2007;60:394‐397. 17561500 10.1093/jac/dkm204

[34] Fihman V , Lartigue MF , Jacquier H , et al. Appearance of aac(6')‐Ib‐cr gene among extended‐spectrum b‐lactamase‐producing Enterobacteriaceae in a French hospital. J Infect. 2008;56:454‐459. 18440645 10.1016/j.jinf.2008.03.010

[35] Kang HY , Tamang MD , Seol SY , Kim J . Dissemination of Plasmid‐mediated qnr, aac(6')‐Ib‐cr, and qepA Genes Among 16S rRNA Methylase producing Enterobacteriaceae in Korea. J Bacteriol Virol. 2009;39:173‐182.

[36] Wang Y , Shen M , Yang J , et al. Prevalence of carbapenemases among high‐level aminoglycoside‐resistant Acinetobacter baumannii isolates in a university hospital in China. Exp Ther Med. 2016;12:3642‐3652. 28101158 10.3892/etm.2016.3828 PMC5228107

